# Single-cell transcriptomics reveals how Shenfu Qiangxin pill ameliorates HFpEF by modulating cardiac cellular heterogeneity

**DOI:** 10.1186/s13020-026-01456-3

**Published:** 2026-07-20

**Authors:** Yakun Yang, Haohao Gao, Zhifei Fu, Weiying Hu, Yijing Li, Haixia Li, Hanqing Lin, Aodi Fan, Ke Yang, Jingyu Ni, Guanwei Fan, Lan Li

**Affiliations:** 1 State Key Laboratory of Modern Chinese Medicine, Key Laboratory of Pharmacology of Traditional Chinese Medical Formulae for the Ministry of Education, Tianjin University of Traditional Chinese Medicine, Tianjin, China; 2 National Clinical Research Center for Chinese Medicine Acupuncture and Moxibustion, First Teaching Hospital of Tianjin University of Traditional Chinese Medicine, Tianjin, China

**Keywords:** Shenfu Qiangxin pill, Single-cell RNA sequencing, HFpEF, Inflammation, Fibrosis, Endothelial dysfunction

## Abstract

**Background:**

Heart failure with preserved ejection fraction (HFpEF) has currently emerged as a predominant and challenging subtype of heart failure, with high morbidity and mortality. However, the efficacy of current therapeutic strategies for HFpEF remains unsatisfactory. The traditional Chinese medicine formulation Shenfu Qiangxin pill (SFQX) ameliorates clinical symptoms in patients with heart failure, but its precise mechanisms for HFpEF remain to be elucidated. This study aimed to investigate the therapeutic potential of SFQX for HFpEF and to elucidate the mechanisms underlying its effects.

**Methods:**

UPLC-Q-TOF-MS/MS analysis was performed to identify the major active ingredients of SFQX. The HFpEF mouse model was established using a high-fat diet and an Nω-Nitro-L-arginine methyl ester hydrochloride (L-NAME) to evaluate the therapeutic efficacy of SFQX. We employed an integrated approach combining single-cell RNA sequencing (scRNA-seq) with functional and molecular validation to characterize SFQX-induced changes in cardiac cellular composition, cell-state remodeling, and tissue-level phenotypes in HFpEF.

**Results:**

We show that SFQX exerts therapeutic effects against HFpEF by coordinately modulating maladaptive cardiac cell subsets. Specifically, SFQX was associated with reprogramming of pathogenic immune cell polarization, normalization of fibroblast state heterogeneity, enhanced endothelial metabolic adaptability, and restoration of lymphatic endothelial homeostasis. These multicellular changes were accompanied by improved cardiac structure and function, reduced fibrosis and inflammation, enhanced lymphatic drainage capacity, and alleviated myocardial edema.

**Conclusion:**

Our findings highlight the ability of SFQX, as a multicomponent agent, to precisely regulate the highly heterogeneous pathology of HFpEF at a network level. This work not only establishes a mechanistic link between holistic principles of traditional medicine and modern biology but also provides a novel theoretical basis for SFQX's efficacy in multifactorial diseases.

**Supplementary Information:**

The online version contains supplementary material available at 10.1186/s13020-026-01456-3.

## Introduction

Heart failure with preserved ejection fraction (HFpEF) is the most common form of heart failure and poses a major unmet clinical challenge due to its high morbidity, mortality, and the lack of effective therapies. The prevalence of HFpEF continues to rise in parallel with population aging and the increasing burden of cardiometabolic disorders, including obesity, hypertension, and diabetes [1]. Unlike heart failure with reduced ejection fraction, for which multiple evidence-based therapies have been established, pharmacological interventions for HFpEF remain limited, underscoring the need for new mechanistic insights and treatment strategies [2]

A defining feature of HFpEF is its biological heterogeneity and complexity. Rather than being driven by a single dominant pathway, HFpEF arises from the convergence of metabolic stress, chronic inflammation, microvascular dysfunction, and extracellular matrix remodeling [3–5]. These pathological processes are executed and sustained by diverse cardiac cell populations, including cardiomyocytes, macrophages, fibroblasts, endothelial cells, and other stromal cells [6–8]. Importantly, HFpEF is increasingly recognized as a disease characterized not only by changes in cellular composition but also by profound alterations in cellular states and functional programs within individual lineages [9]. Therefore, dysregulated cellular heterogeneity, reflected by the expansion of maladaptive cell subsets and the loss of homeostatic populations, has emerged as a central determinant of disease progression.

Single-cell RNA sequencing (scRNA-seq) has provided direct evidence for this concept by mapping the cellular and molecular landscape of HFpEF at single-cell resolution. For instance, scRNA-seq of CD45⁺ cardiac cells in HFpEF showed a heterogeneous macrophage response to metabolic stress, with a dynamic shift between resident (reparative) and recruited (pro-inflammatory) subsets [10]. Fibroblasts display marked heterogeneity, characterized by distinct profibrotic activation states and altered differentiation trajectories [11]. In contrast, endothelial cells exhibit metabolic reprogramming and functional diversification that are closely associated with microvascular dysfunction [8]. However, whether pharmacological interventions can therapeutically reshape this pathological cellular heterogeneity remains largely unknown.

Traditional Chinese medicine (TCM) is characterized by its multi-component and multi-target therapeutic properties, which enable coordinated regulation of multiple biological processes [12, 13]. From a systems biology perspective, TCM formulations may be particularly well suited to diseases driven by complex cellular networks and heterogeneous cell-state alterations [13]. Instead of acting on isolated molecular targets, TCM therapies are hypothesized to modulate disease-associated cellular programs across multiple cell types, thereby restoring tissue-level homeostasis. Nevertheless, direct experimental evidence demonstrating how TCM formulas regulate cellular heterogeneity in complex cardiac diseases such as HFpEF is still lacking.

Shenfu Qiangxin pill (SFQX) is a modern standardized formula based on TCM theory. It is composed of *Ginseng Radis Et Rhizoma* (Renshen), processed *Aconiti Lateralis Radix Praeparata* (Fuzi), *Rhei Radix et Rhizoma* (Dahuang), *Lepidii Semen* (Tinglizi), *Mori Cortex* (Sangbaipi) and *Polyporus* (Zhuling). Grounded in the traditional principle of “tonifying Qi and boosting Yang, strengthening the heart and promoting fluid excretion”, SFQX is clinically applied to treat chronic heart failure (CHF) [14, 15] characterized by symptoms such as dyspnea, edema, and reduced exercise tolerance. Modern pharmacological studies support these classical concepts, confirming that SFQX exerts cardioprotective effects by reducing fluid retention, correcting electrolyte imbalances, and suppressing myocardial inflammation, autophagy, and apoptosis [14, 16]. However, its efficacy and underlying mechanisms in HFpEF remain poorly understood.

In this study, we employed scRNA-seq integrated with functional and molecular validation to investigate the effects of SFQX in a murine model of HFpEF. We aimed to determine whether SFQX alleviates HFpEF by reprogramming disease-associated cellular heterogeneity across multiple cardiac cell lineages, including immune, stromal, and endothelial populations. Our findings demonstrate that SFQX coordinately modulates maladaptive cell subsets, restores homeostatic cellular states, and improves cardiac structure and function. This work provides single-cell–level evidence that a multi-component therapy can therapeutically reshape pathological cellular heterogeneity in HFpEF, offering a mechanistic framework for the development of systems-oriented treatments for complex heart failure syndromes.

## Materials and methods

### Animals

All experiments were approved by the Animal Care Committee of Tianjin University of Traditional Chinese Medicine (Approval No. TCM-LAEC2024135F1127). Briefly, male C57BL/6 mice (22 ± 2 g) aged 6–8 weeks were purchased from the Vital River Laboratory Animal Technology Co., Ltd. (Beijing, China, certificate number: SCXK [Jing] 2021–0006). All mice were housed under standard specific pathogen-free (SPF) conditions, maintained on a 12-h light/dark cycle at a temperature of 24 °C.

### Experimental design

To induce the HFpEF phenotype, mice were fed a high-fat diet (HFD; Research Diets, D12492), while concurrently receiving drinking water supplemented with Nω-Nitro-L-arginine methyl ester hydrochloride (L-NAME; Sigma-Aldrich, #N5751) at a concentration of 0.5 g/L (pH adjusted to 7.4) [17]. Following successful HFpEF induction, mice were administered empagliflozin (EMPA, 10 mg/kg) or SFQX at low (SFQX-L, 2.25 g/kg) or high (SFQX-H, 4.5 g/kg) doses via oral gavage daily for four weeks, as depicted in Fig. 2A. EMPA was obtained from Boehringer-Ingelheim (Ingelheim am Rhein, Germany) and SFQX from Tianjin Pharmaceutical Da Rentang Group Co., Ltd. (Tianjin, China). Finally, mice were sacrificed under deep anesthesia, and blood, heart, and lung tissues were collected for subsequent analysis.

### UPLC-Q-TOF-MS/MS analysis

Chromatographic separation was performed on a Waters Acquity UPLC system equipped with an HSS T3 column (2.1 × 100 mm, 1.8 μm). The mobile phase consisted of (A) water containing 0.1% formic acid and (B) acetonitrile, delivered at a flow rate of 0.3 mL/min. The column temperature was maintained at 35 °C, and the injection volume was 2 μL. The gradient elution program was as follows: 0–24 min, 5% to 100% B; 24–26.5 min, 100 B; 26.5–27 min, 100% to 5% B; 27–30 min, 5% B. The scanning mode was Full Scan/Data Dependent Secondary Scan (Full MS/dd-MS2), and the mass spectrometry scanning range was m/z 100–1500, with collision energy gradients of 10, 30, and 50 eV. The data acquired using Xcalibur 4.1 software. The raw data files were processed using CD3.0 software for characteristic peak extraction, followed by compound identification using mzCloud, mzVault, and self-built databases.

### Preparation of single-cell suspension

Briefly, freshly collected cardiac tissue was washed with culture medium, minced into small fragments, and digested with an enzymatic solution at 37 °C for 30 min. The digested cell suspension was filtered through a 40 μm cell strainer, centrifuged at 300 × *g* for 7 min at 4 °C, and the resulting single-cell suspension was collected. CD45^+^ immune cells were then enriched from the suspension using mouse CD45 micro beads. Concurrently, the other half of the tissue was lysed, filtered, and centrifuged to prepare a single-nucleus suspension. Finally, the single-cell suspension and the single-nucleus suspension were mixed at a 1:1 ratio based on cell/nucleus count.

### scRNA-seq and data processing

Library preparation and loading were performed according to the manufacturer's instructions of the 10 × Genomics Chromium Next GEM Single Cell 3ʹ Reagent Kits v3.1 (Product No. PN-1000268). The constructed libraries were subjected to high-throughput sequencing using the BGI DNBSEQ-T7 PE100 platform. Raw expression matrices from distinct samples were merged using Seurat, followed by quality control to filter out low-quality cells and technical noise. Subsequently, normalization was performed via the LogNormalize method, with identification of highly variable genes and principal component analysis (PCA) for dimensionality reduction. RNA contamination was eliminated using decontX, while doublets were detected and removed through DoubletFinder.

Batch effects were corrected with Harmony, followed by clustering analysis employing the Louvain algorithm and visualization via Uniform Manifold Approximation and Projection (UMAP) and t-distributed Stochastic Neighbor Embedding (t-SNE). Marker genes for each cluster were screened, and cell types were manually annotated. Differences in cell composition between groups were compared, and differentially expressed genes (DEGs) were identified using the Wilcoxon test, with pathway activity scoring conducted via irGSEA. Transcription factor (TF) activity was analyzed by integrating the DoRothEA database with the VIPER algorithm. The starting point of pseudotime differentiation was determined using CytoTRACE2, and the developmental differentiation trajectory of cells was constructed with Monocle2/3.

Cell–cell communication analysis was performed using CellChat. Separate CellChat objects were constructed for the control, HFpEF, and SFQX groups, with CellChatDB.mouse selected as the species-specific ligand-receptor database. Following standard preprocessing, gene expression data were projected onto the mouse protein–protein interaction network to enhance the detection sensitivity of low-abundance signals. Communication probabilities were then computed at the ligand-receptor level. Cell types containing fewer than 10 cells were filtered out, after which signals were aggregated to the pathway level and the aggregated communication network was constructed. Signaling network centrality for each cell type was calculated using the netP slot from the CellChat object. Finally, the three group-specific CellChat objects were merged using the mergeCellChat function for comparative analysis.

### Echocardiography and Doppler imaging

Cardiac function was assessed noninvasively using a high-frequency ultrasound imaging system (VINNO 6 LAB, Suzhou, China). Mice were anesthetized via inhalation of isoflurane (induction at 2–5%, maintenance at 1–1.5%) delivered in oxygen. Standard two-dimensional and M-mode images were acquired from the parasternal short-axis view to measure left ventricular ejection fraction (LVEF). Diastolic function was assessed using both pulsed-wave Doppler (PWD) and tissue Doppler imaging (TDI) in the apical four-chamber view to measure trans-mitral blood flow velocities and myocardial velocities, respectively. Following the examination, the mice recovered from anesthesia without complications.

### Tail-cuff blood pressure recordings

Blood pressure was assessed in conscious mice using a tail-cuff system (BP-2000 Series II, Visitech Systems, USA). Measurements were conducted once the animals were calm and stable. A series of cuff inflations/deflations were performed, and the average of multiple stable readings (typically from at least 3 successful cycles per session over several days) was recorded as the final blood pressure value.

### Exercise exhaustion test

Briefly, exercise capacity was assessed using a treadmill (Zhishuduobao, Beijing, China) exhaustion test after a 3-day acclimation. Mice ran on an inclined treadmill with a progressively increasing speed protocol until exhaustion, defined by specific contact with a stimulus grid. The total running distance were recorded as primary outcomes.

### Whole-mount immunohistochemical staining

Mouse hearts were collected and fixed overnight in 4% paraformaldehyde at 4 °C. Tissue clearing was performed using a commercial tissue clearing kit (Cat#NH-CR-230701, Shanghai, China). Briefly, samples were incubated with the primary antibody (anti-LYVE1, Abcam, Cat# ab14917, 1:200) in a shaker at 37 °C for 7 days, followed by incubation with the secondary antibody (Abcam, Cat# ab150077, 1:500) for an additional 5 days. Finally, the cleared heart tissues were subjected to three-dimensional (3D) imaging using an LS18 light-sheet microscope (Nuohai Life Science Co., Ltd., China) and rendered with Amira software (Thermo Fisher Scientific, USA).

### Evans blue staining of the heart

The fluid drainage capacity of cardiac lymphatic vessels was evaluated using Evans blue staining. Mice were anesthetized with tribromoethanol prior to surgery. After surgical exposure of the cardiac apex, 10 µL of Evans blue dye (20 mg/mL) was injected into the same site at the apex of each mouse, followed by wound closure. Four hours later, the mice were re-anesthetized, and their hearts were harvested. Residual Evans blue dye was then extracted using formamide. The level of residual dye in cardiac tissue was measured by absorbance spectrophotometry at 610 nm, as previously described [18].

### Biochemical determination

The concentrations of heart failure markers in plasma samples were measured using commercial ELISA kits according to the manufacturers’ instructions. The atrial natriuretic peptide (ANP) was detected using the Mouse ANP ELISA kit (Cat No. E-EL-M0166, Elabscience, China). The N-terminal pro-BNP (NT-proBNP) was detected using the Mouse NT-proBNP ELISA kit (Cat No. E-EL-M0834, Elabscience, China).

### Histological analysis

Heart tissues were collected and fixed in 4% paraformaldehyde, followed by embedding in paraffin. Tissue sections were cut at a thickness of 5 μm. Sections were stained with hematoxylin and eosin (H&E) to assess general morphology and cardiac hypertrophy. Collagen deposition was evaluated using Masson’s trichrome and Sirius red staining, while wheat germ agglutinin (WGA) was employed to determine cardiomyocyte cross-sectional area. Stained sections were imaged using a fluorescence microscope (Leica THUNDER, Germany).

### Intraperitoneal glucose tolerance test

An intraperitoneal glucose tolerance test was performed to assess glucose homeostasis. Mice were fasted for 12 h prior to the test. After the fasting period, baseline blood glucose (0 min) was measured from the tail vein using a glucometer. Subsequently, a sterile D-glucose solution (2 g/kg body weight) was administered via intraperitoneal injection. Blood glucose levels were then monitored at specified time points (e.g., 15, 30, 60 and 120 min) post-injection.

### Flow cytometry detection

Peripheral blood samples were treated with 10 mL of red blood cell lysis buffer for 20 min at 4 °C to remove erythrocytes. After lysis, the cells were pelleted by centrifugation at 1000 rpm for 10 min. The supernatant was discarded, and the cell pellet was resuspended in 100 μL of staining buffer. The cells were then stained with the following fluorochrome-conjugated antibodies: anti-mouse CD45-PerCP-Cy5.5, anti-mouse CD11b-APC, anti-mouse LY6C-APC-CY7, and anti-mouse CCR2-PE (Biolegend, USA), followed by incubation at 37 °C for 30 min in the dark. Finally, the pellet was resuspended in 800 μL of staining buffer, filtered through a 70-μm cell strainer, and subjected to flow cytometry analysis.

### High throughput assays for serum inflammatory cytokines

Several serum inflammatory cytokines were detected by the LEGENDplex^™^ Mouse Inflammation Panel (Cat. No. 740446, Biolegend). Briefly, standards and plasma samples (diluted 1:2) were added into a “V-bottom” 96-well plate. The plate was then incubated on a plate shaker at 800 rpm for 2 h at room temperature. Subsequently, 25 μL of detection antibody was added to each well and beads were resuspended in 200 μL of wash buffer for immediate acquisition on a flow cytometer, as described previously [19].

### Real-time quantitative PCR analysis

Total RNA was extracted from heart tissues using TRIzol reagent (Cat. No. ER501-01, TransGen Biotech, Beijing). Quantitative real-time PCR was then performed using ChamQ Blue Universal SYBR qPCR Master Mix (Cat. No. Q312, Vazyme, Nanjing) on a QuantStudio 6 Real-Time PCR System (Thermo Fisher Scientific, USA). The sequences of the gene-specific primers used are listed in Table S1. The relative mRNA expression levels of target genes were calculated using the comparative 2^− ΔΔCt^ method, with β-actin serving as the control for normalization.

### Western blotting

Protein was extracted from cardiac tissues homogenized in RIPA lysis buffer supplemented with protease inhibitors. The homogenates were centrifuged at 12,000 × *g* for 15 min at 4 °C to collect the supernatant. Subsequently, 30 μg protein were denatured in 5 × SDS loading buffer by heating at 95 °C for 10 min. The denatured proteins were separated by electrophoresis on SDS-polyacrylamide gels and then transferred onto PVDF membranes (Millipore, USA). After blocking, membranes were incubated overnight at 4 °C with the primary antibody, included Collagen I (ab34710, Abcam, 1:2000), Collagen III (HA720050, HUABIO, 1:1000), α-SMA (#19,245, CST, 1:1000), Vegfa (#26,157–1-AP, 1:1500, Proteintech), Vegfr-2 (F3049, Selleck, 1:1000) and GAPDH (#60,004-1-Ig, 1:100,000, Proteintech). After washing, the membranes were incubated with appropriate HRP-conjugated secondary antibodies for 1 h at room temperature. Protein bands were visualized by ChemDoc Gel Imaging System and assessed by Image J software.

### Statistical analysis

All quantitative data are presented as the mean ± SD. Statistical analyses were performed using GraphPad Prism 7.0 software. Multiple comparisons were performed using one-way ANOVA and Dunnett's multiple comparisons test. The student's t-test was used to compare between two groups. A* p*-value of less than 0.05 was considered statistically significant.

## Results

### Characterization of major active ingredients in SFQX

**Fig. 1:**
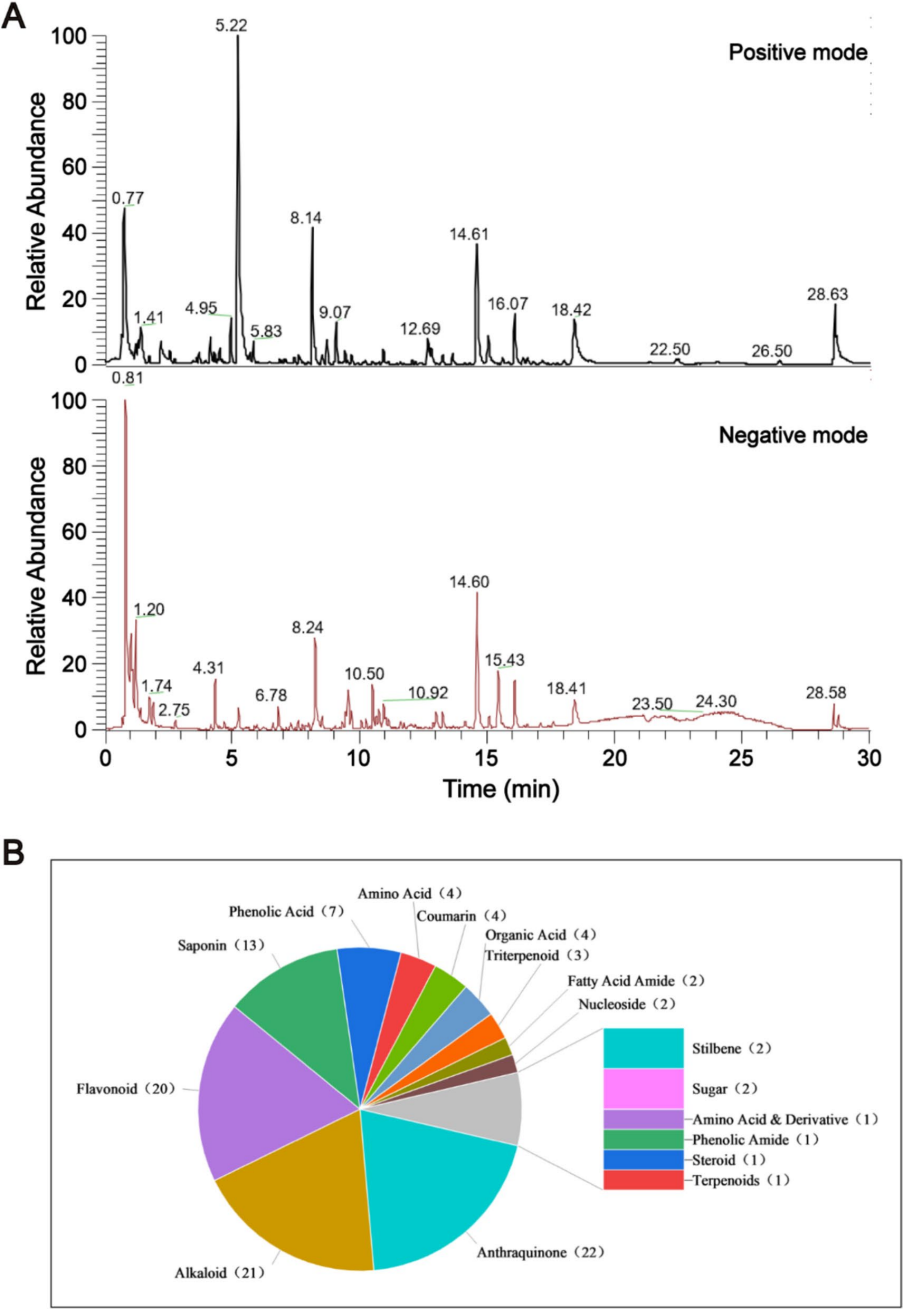
The UPLC-Q-TOF–MS/MS analysis of SFQX. **A** Chromatogram in positive (upper) and negative (lower) ion modes. **B** Numbers of different types of compounds

Figure 1A presents the UHPLC-MS base-peak chromatogram (BPC) profiles of SFQX in both the positive-ion and negative-ion conditions. Compound identification was performed based on accurate molecular mass, analysis of secondary MS/MS fragment ions, and database searches, including PubMed, ScienceDirect, ChemSpider, and a custom-built in-house library. Using this approach, a total of 110 chemical compounds were identified in SFQX pills provisionally. These compounds were categorized into five major groups, categorized as 22 anthraquinones, 21 alkaloids, 20 flavonoids, 13 saponin, and 7 phenolic acids (Fig. 1B). Table S2 details all 110 identified compounds.

### SFQX attenuates the clinical manifestations exhibited by HFpEF mice

**Fig. 2:**
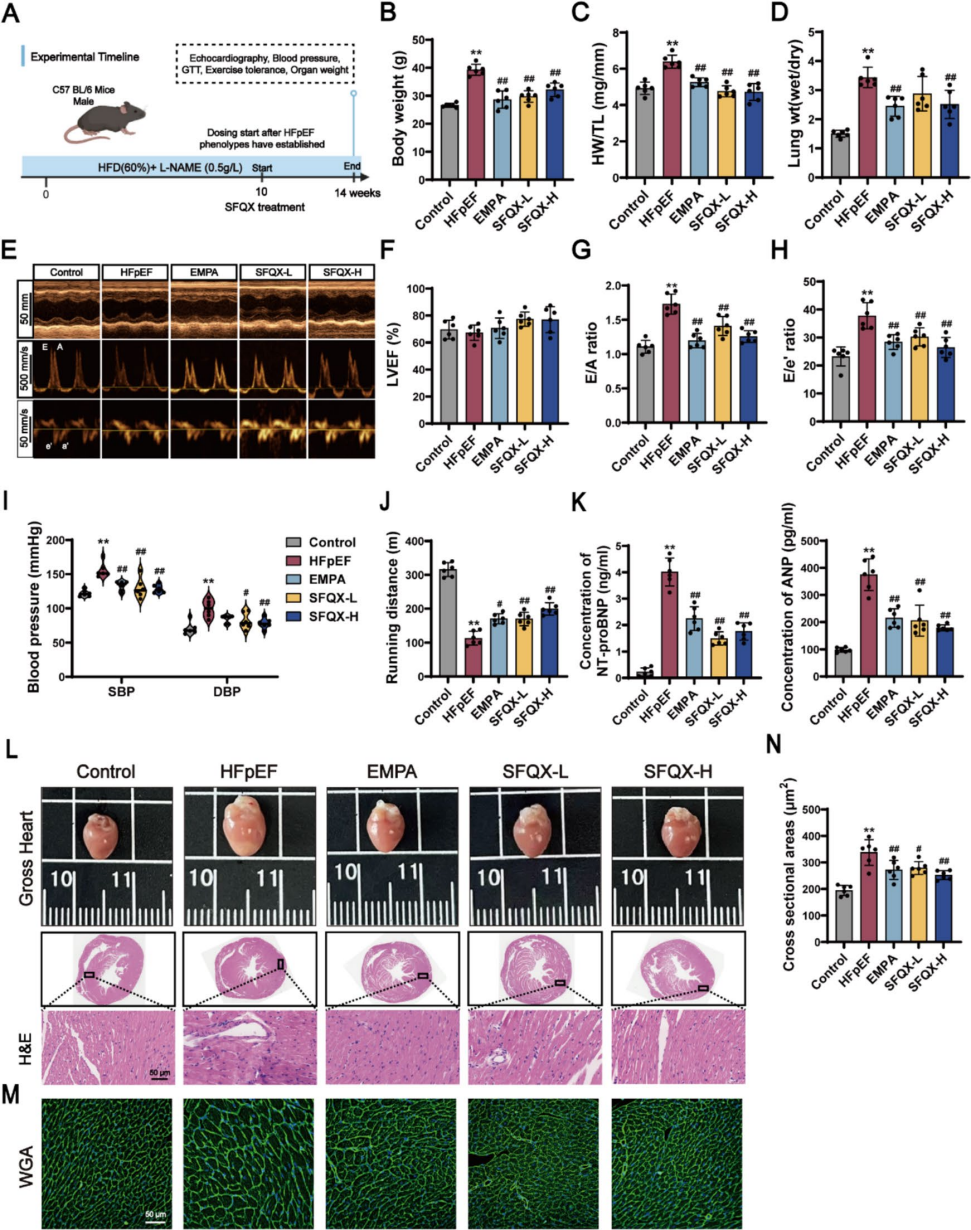
SFQX attenuates the clinical manifestations exhibited by HFpEF mice. **A** Schematic diagram of HFpEF model and drug intervention. **B** Body weight. **C** Heart weight/tibia length (HW/TL) ratio. **D** Lung wet-to-dry weight ratio. **E** Representative echocardiographic images (M-mode, pulse-wave Doppler, and tissue Doppler). Quantitative analysis of **F** LVEF (%), **G** E/A, and **H** E/e’. **I** Blood pressure. **J** Running distance. Concentration of **K** NT-proBNP and ANP. **L** Representative images of gross cardiac morphology and H&E. (n = 3). **M** Representative images of WGA staining. (scale bar = 50 μm). **N** Quantification of cardiomyocyte cross-sectional area in each group. 6 cardiomyocytes per mouse (n = 3 mice per group). Data are presented as mean ± SD (n = 6). *p < 0.05, **p < 0.01 vs. Control group. ^*#*^*p* < 0.05, ^*##*^*p* < 0.01 vs. HFpEF group

To investigate the role of SFQX in HFpEF, we assessed the HFpEF-associated clinical manifestations elicited in mice using two different doses of SFQX, with EMPA included as a positive control. As shown in Fig. 2B–D, HFD + L-NAME feeding (HFpEF) induced significant increases in body weight, the heart weight-to-tibia length (HW/TL) ratio, and the lung wet-to-dry weight ratio, all of which were ameliorated by SFQX and EMPA treatment. Importantly, SFQX treatment ameliorated diastolic dysfunction in HFpEF mice, as evidenced by reduced E/A and E/e' ratios, whereas LVEF remained unchanged across all groups, underscoring the specificity of the model for preserved ejection fraction (Fig. 2E-H). Moreover, SFQX treatment significantly reduced both systolic and diastolic blood pressure, increased the exercise distance, and lowered the levels of heart failure biomarkers (ANP and NT-proBNP) in HFpEF mice (Fig. 2I–K). Gross cardiac morphology and WGA staining revealed that both SFQX and EMPA significantly attenuated cardiac hypertrophy in HFpEF mice (Fig. 2M), which was consistent with the findings from the HW/TL ratio. Furthermore, H&E staining revealed that SFQX and EMPA treatment alleviated histopathological injuries in HFpEF mice, including nuclear aggregation, myocyte disarray, and inflammatory cell infiltration (Fig. 2L). Taken together, these results demonstrate that SFQX mitigates structural and functional cardiac abnormalities in HFpEF mice.

### SFQX ameliorates cardiac metabolic stress in HFpEF mice

**Fig. 3:**
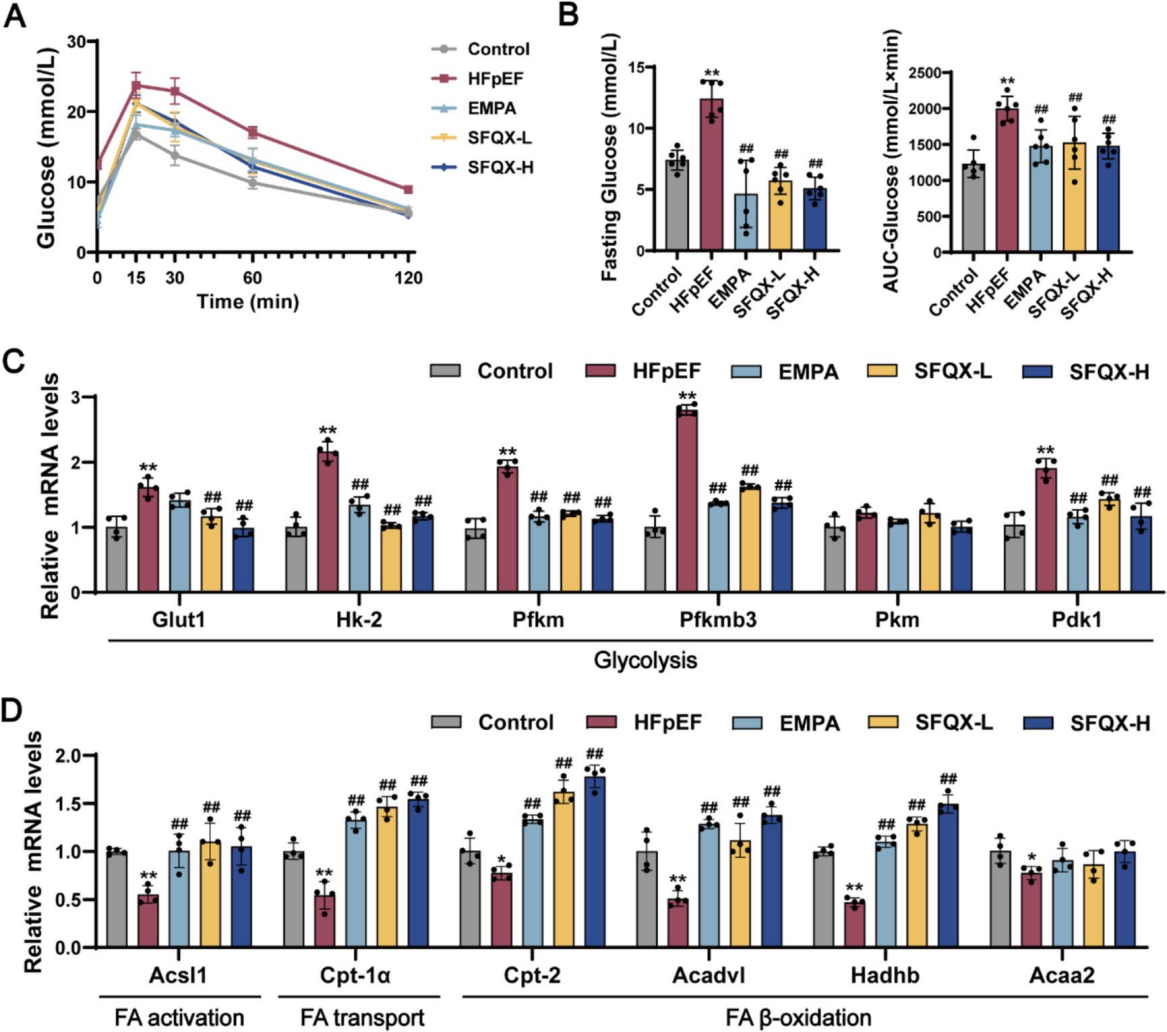
SFQX ameliorates cardiac metabolic stress in HFpEF mice. **A** Glucose tolerance test in each group. **B** Quantitative analysis of fasting glucose and area under the curve (AUC) for the glucose tolerance test, n = 6. **C** mRNA expression of glycolysis-related genes in the heart, n = 4. **D** mRNA expression levels of genes related to fatty acid activation, transport, and β-oxidation in each group, n = 4. Data are presented as mean ± SD. *p < 0.05, **p < 0.01 vs. Control group. ^*#*^*p* < 0.05, ^*##*^*p* < 0.01 vs. HFpEF group

Cardiac metabolic stress is a key driver of HFpEF, characterized by impaired myocardial energy metabolism that results in insufficient energy production [20]. We next investigated whether the therapeutic benefits of SFQX are associated with the amelioration of myocardial metabolic stress. Our results demonstrated that a significant improvement in glucose tolerance and fasting blood glucose recovery was observed in HFpEF mice treated with SFQX or EMPA relative to the HFpEF group (Fig. 3A, B). Consistent with the metabolic remodeling observed in HFpEF [21], qPCR analysis revealed a transcriptional profile indicative of a shift toward aerobic glycolysis. Specifically, the mRNA levels of key glycolytic genes, including *Glut1*, *Hk-2*, *Pfkm*, and *Pfkfb3*, were significantly upregulated in HFpEF hearts. Notably, the increased expression of *Pdk1* in combination with unaltered *Pkm *expression suggests an impaired coupling between glycolysis and glucose oxidation. Critically, SFQX treatment markedly attenuated these alterations, normalizing the expression of *Glut1*, *Hk-2*, *Pfkm*, and *Pfkfb3*, and potently reversing the upregulation of *Pdk1* (Fig. 3C). Analysis of fatty acid oxidation genes revealed a coordinated downregulation in the HFpEF myocardium, affecting *Acsl1*, *Cpt1a*, *Cpt2*, *Acadvl*, and *Hadhb*, all of which were restored by SFQX treatment. In contrast, the expression of *Acaa2* remained moderately altered in HFpEF and was unaffected by SFQX (Fig. 3D). In summary, SFQX ameliorates the pathological metabolic remodeling in HFpEF by restoring metabolic flexibility, which corrects substrate utilization and enhances cardiac energy production.

### Single-cell atlas of the heart in HFpEF mice revealed by scRNA-Seq

**Fig. 4:**
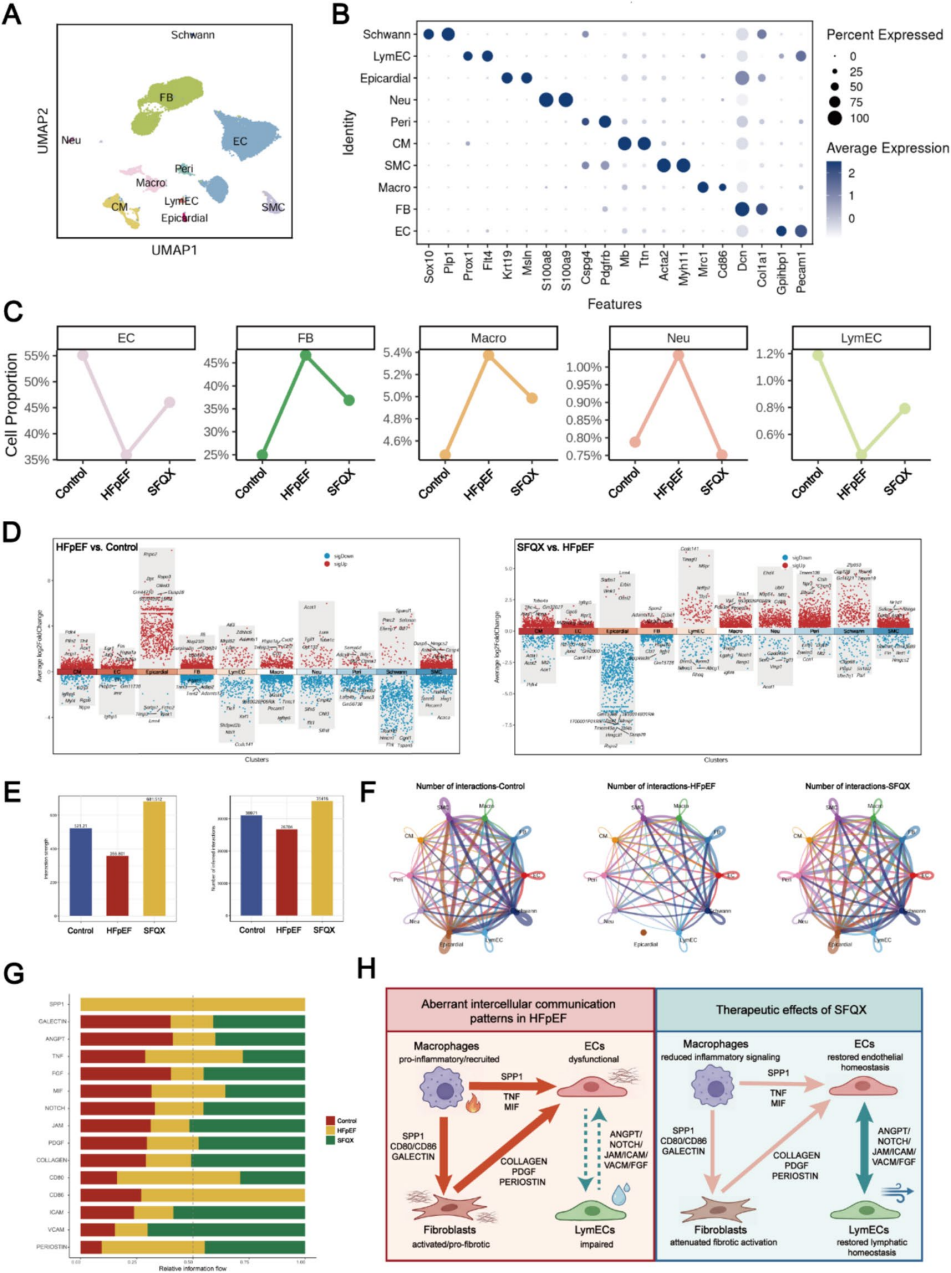
Single-cell atlas of the heart in HFpEF mice. **A** Visualization of cell populations via Uniform Manifold Approximation and Projection (UMAP), with groups color-coded. **B** Projection of cell type-specific marker gene expression onto a bubble chart. **C** Proportion of main cell types in Control, HFpEF and SFQX group. **D** Comparative analysis of gene expression for all cell types: HFpEF vs. Control and SFQX vs. HFpEF. **E** The strength and number of interactions in the Control, HFpEF, and SFQX groups. **F** The intercellular interaction network in the three groups. Edge width represents the interaction strength, and the edges are color-coded based on the signal source. **G** Relative information flow for each signaling pathway in the three groups, defined as the sum of communication probabilities across all subpopulation pairs. **H** Schematic diagram of SFQX restoring intercellular communication in HFpEF

To elucidate the impact of SFQX on cellular composition and transcriptional profiles in cardiac tissue, we constructed scRNA-seq atlases for the Control, HFpEF, and SFQX-treated groups. After filtration and quality control, 23,516 cells were classified into ten major cell types according to specific markers: endothelial cells, fibroblasts, macrophages, smooth muscle cells, cardiomyocytes, pericytes, neutrophils, epicardial cells, lymphatic endothelial cells, and schwann cells (Fig. 4A, B). To characterize the dynamics of cellular composition, we analyzed cell-type proportions across the three groups. Notably, SFQX treatment successfully rescued the altered cell abundances observed in the HFpEF group. Specifically, SFQX significantly reduced the proportions of fibroblasts, macrophages, and neutrophils, while increasing the endothelial cell population, particularly in the lymphatic endothelial cell population (Fig. 4C). Subsequently, analysis revealed the DEGs for all cell types through comparisons between the HFpEF and control groups, as well as between the SFQX and HFpEF groups. (Fig. 4D, Table S3). Taken together, these results reveal the cell-type-specific effects of SFQX in HFpEF mice, thereby advancing a more comprehensive understanding of its therapeutic role.

### SFQX reverses aberrant intercellular communication pattern in HFpEF

CellChat was used to infer cell–cell communication across cell types and groups. Compared with the control group, the HFpEF group exhibited a marked reduction in overall interaction strength and interaction number, indicating suppressed multicellular coordination in diseased myocardium. SFQX treatment partially restored these parameters, suggesting recovery of intercellular connectivity at the tissue level (Fig. 4E). We next focused on key pathological cell types identified by scRNA‑seq, including macrophages, fibroblasts, endothelial cells, and lymphatic endothelial cells. In HFpEF, communication among these lineages shifted toward a maladaptive inflammatory/fibrotic network. Specifically, macrophage-to-fibroblast and macrophage-to-endothelial signaling was strengthened, whereas endothelial- and lymphatic-related homeostatic communication was weakened (Fig. 4F). This pattern supports the view that chronic inflammation, stromal activation, and vascular dysfunction in HFpEF are not isolated but linked through an abnormal multicellular signaling program.

To further define how SFQX regulates this network, we examined representative signaling pathways relevant to macrophage, fibroblast, and endothelial remodeling. Three-group comparison of relative information flow showed that HFpEF shifted intercellular communication toward inflammatory and pro-fibrotic programs, with prominent enrichment of SPP1, CD86, TNF, GALECTIN, COLLAGEN and PERIOSTIN. These changes align with enhanced macrophage activation, fibroblast stimulation, and matrix remodeling in HFpEF. SFQX attenuated this inflammatory/fibrotic signaling and strengthened endothelial/lymphatic homeostatic programs, including ANGPT, NOTCH, ICAM, VCAM, and FGF signaling (Fig. 4G). These data indicate that SFQX redirects communication from an inflammatory/pro-fibrotic to a homeostatic state, not merely amplifying global signals (Fig. 4H). Consistently, focused summary of incoming/outgoing signaling patterns showed that HFpEF reinforced a macrophage-centered inflammatory/fibrotic hub linking macrophages, fibroblasts, and endothelial cells, while SFQX attenuated these maladaptive interactions and restored endothelial/lymphatic homeostasis (Fig. S1).

### SFQX modulates macrophage subsets to mitigate inflammatory responses

**Fig. 5:**
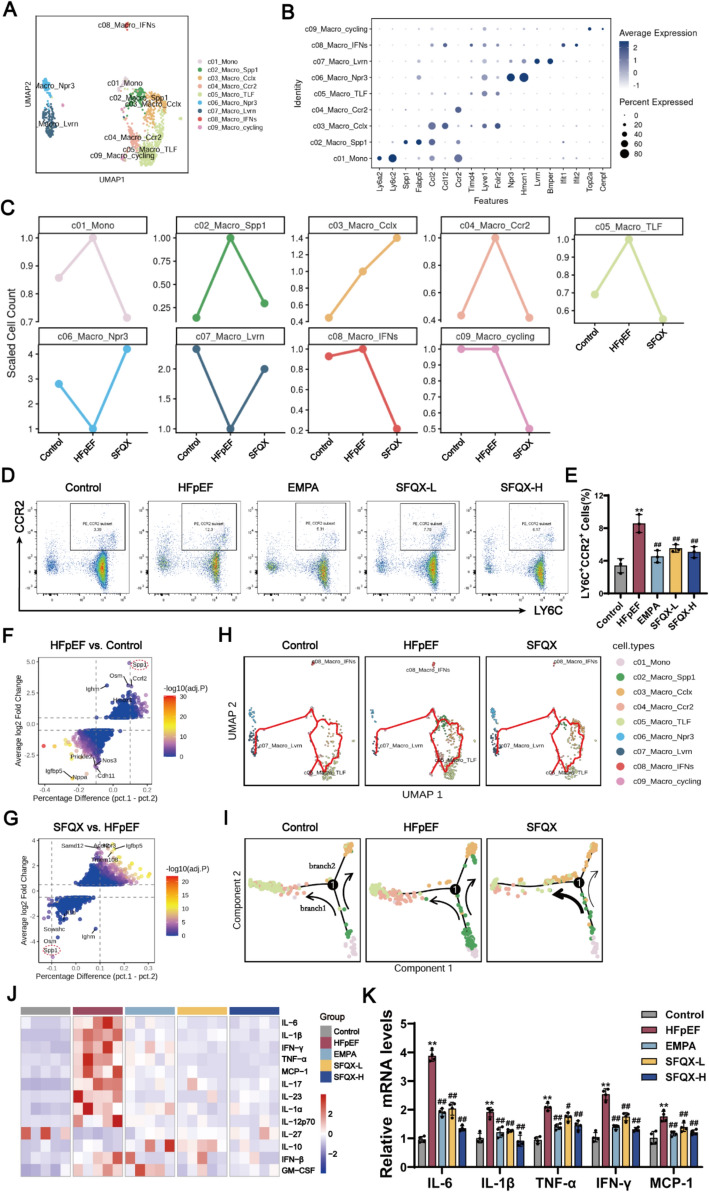
SFQX modulates macrophage subsets to mitigate inflammatory responses. **A** UMAP embedding of macrophages, colored by manually annotated subsets. **B** Marker genes for each macrophage cluster. **C** Abundance of macrophage subsets in Control, HFpEF, and SFQX groups. **D** Flow cytometry analysis, and **E** quantification of LY6C^+^/CCR2^+^ cells, respectively, n = 3. Identification of differentially expressed genes (DEGs) in macrophages: **F** HFpEF versus Control, and **G** SFQX treatment versus HFpEF. **H** Inference of the pseudotime trajectories of macrophages, color-coded as per the cell subsets. **I** Analysis of cellular state proportions along pseudotime trajectories across three groups. **J** LEGENDplex™ quantification of inflammatory cytokines in serum, n = 6. **K** Inflammatory factor mRNA expression levels, n = 4. Data are presented as mean ± SD. *p < 0.05, **p < 0.01 vs. Control group. ^*#*^*p* < 0.05, ^*##*^*p* < 0.01 vs. HFpEF group

Dysregulated inflammation and immune responses are now recognized as central drivers in the pathophysiology of HFpEF [7]. Macrophages, as key components of the innate immune system, can polarize into pro- or anti-inflammatory phenotypes in response to local environmental cues [22]. Therefore, we explored the key macrophage subsets modulated by SFQX in HFpEF by reclustering all macrophages. Unbiased clustering resolved the macrophage lineage into nine distinct subsets, which exhibited individual gene expression profiles (Fig. 5A, B). They were primarily identified as classical monocyte precursors (c01_Mono, marked by Ly6c, Ly6a), reparative macrophages (c02_Macro_Spp1, marked by Spp1), pro-inflammatory macrophages (c03_Macro_Cclx, highly secreting members of the CC chemokine family), monocyte-recruited macrophages (c04_Macro_Ccr2, marked by Ccr2), tissue-resident reparative macrophages (c05_Macro_TLF, marked by Timd4, Lyve1, and Folr2), and interferon-responsive macrophages (c08_Macro_IFNs, marked by Ifit2 and Ifit1). We found that SFQX reduced monocyte-derived macrophages, as evidenced by a decreased cellular ratio of the C01 to C04 clusters (Fig. 5C). These cells differentiate into pro-inflammatory macrophages, thereby serving as critical drivers of myocardial fibrosis and diastolic dysfunction [23]. Consistent with this, flow cytometry results demonstrated that SFQX markedly decreased the proportion of LY6C⁺CCR2⁺ cells in the peripheral blood of HFpEF mice (Fig. 5D, E).

Analysis of DEGs in macrophages revealed distinct transcriptional profiles among the groups (Fig. 5E, F). A key finding was the significant upregulation of SPP1 in the HFpEF group (Fig. 5F, G). SPP1 encodes osteopontin, a matricellular protein that serves as a central hub connecting inflammatory responses, immune cell recruitment, and fibrosis, whose levels are known to be elevated in HFpEF [11, 24]. Our results are in line with this observation. Importantly, this dysregulation was effectively reversed by SFQX treatment (Fig. 5F, G). Further pseudotime analysis indicated that SFQX reshaped the differentiation trajectory of macrophages, favoring a path toward branch 1 over branch 2. This shift corresponds to a phenotypic evolution from a pro-inflammatory signature toward one resembling resident macrophages (Fig. 5H, I). Resident macrophages are critical for maintaining tissue homeostasis [25]. Inducing a phenotypic shift toward this state can suppress excessive inflammation, promote its resolution, and thereby alleviate myocardial injury [26]. Similarly, our results confirmed that SFQX treatment significantly lowered pro-inflammatory cytokine levels while increasing anti-inflammatory cytokine levels in the serum and cardiac tissue of HFpEF mice (Fig. 5J, K). Collectively, these results suggest that SFQX ameliorates cardiac injury by coordinating the regulation of macrophage polarization and inflammation.

### SFQX modulates fibroblast subsets to reduce cardiac fibrosis

**Fig. 6:**
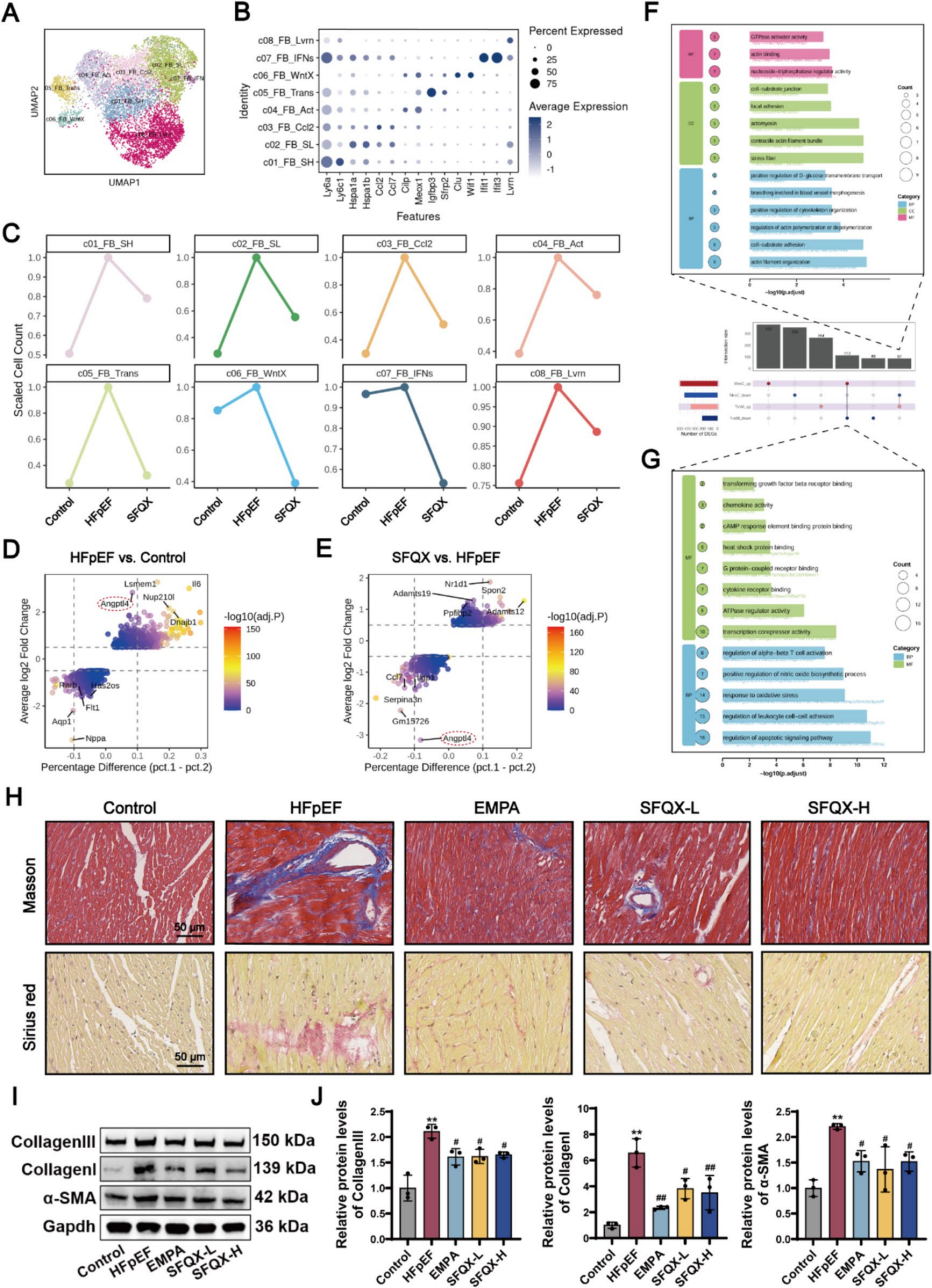
SFQX modulates fibroblast subsets to reduce cardiac fibrosis. **A** UMAP embedding of fibroblasts (FBs), colored by manually annotated subsets. **B** Marker genes for each FB cluster. **C** Abundance of FB subsets in Control, HFpEF, and SFQX groups. Identification of DEGs in FBs: **D** HFpEF versus Control, and **E** SFQX treatment versus HFpEF. **F**, **G** Bio-functional analysis of the DEGs. **H** Representative images of Masson and Sirius red. **I**, **J** Western blot analysis of Collagen III, Collagen I, and α-SMA protein expressions. Data are presented as mean ± SD (n = 3). *p < 0.05, **p < 0.01 vs. Control group. ^*#*^*p* < 0.05, ^*##*^*p* < 0.01 vs. HFpEF group

Fibroblast activity is directly linked to the cardiac fibrosis that characterizes human HFpEF [27]. Based on their distinct gene expression patterns, 8,631 FB cells were categorized into eight distinct clusters (Fig. 6A, B). The c01_FB_SH cluster, characterized by high expression of the Ly6a gene, represents a reserve subpopulation within the fibroblast pool. The c03 cluster, identified as a chemokine-secreting fibroblast subset, exhibits elevated levels of the cytokine Ccl2. Notably, this subset also shows high expressions of Timp1 and Serpine1, which are known to significantly enhance extracellular matrix (ECM) deposition and exacerbate fibrosis progression [28]. The c04_FB_Act cluster is defined as an activated fibroblast subset, marked by high expression of the fibrotic markers Cilp and Meox1 [29]. Notably, SFQX treatment ameliorated the dysregulation observed in all fibroblast clusters from the HFpEF group, albeit to varying degrees (Fig. 6C). Next, we analyzed DEGs between the HFpEF group and the control group, as well as between the SFQX group and the HFpEF group (logFC > 0.5, min.pct > 0.1) (Fig. 6D, E). In the HFpEF group, upregulated genes included Il-6, Angptl4, and Dnajb1, whereas genes downregulated by SFQX treatment included Angptl4, Ccl7, Iigp1, and Serpina3n. Angptl4 (Angiopoietin-like 4), a lipoprotein lipase inhibitor, contributes to the pathogenesis of HFpEF when its expression is elevated [11]. We observed that SFQX treatment effectively downregulated the level of Angptl4. Furthermore, a bio-functional analysis of the common targets regulated by the drug for disease intervention was conducted, as shown in Fig. 6F, G. The results indicated that SFQX modulates the cytoskeletal and cell adhesion systems, including focal adhesions, stress fibers, and actomyosin structures. This process is closely associated with its anti-fibrotic effects. Finally, histopathological examination with Masson and Sirus red staining demonstrated that SFQX significantly attenuated both interstitial and perivascular fibrosis in HFpEF mice (Fig. 6H). In addition, SFQX treatment reduced the expression levels of Collagen I, Collagen III, and α-SMA, indicating its role in alleviating HFpEF-induced cardiac fibrosis (Fig. 6I, J).

### SFQX modulates endothelial cell subsets to improve angiogenic disorders

**Fig. 7:**
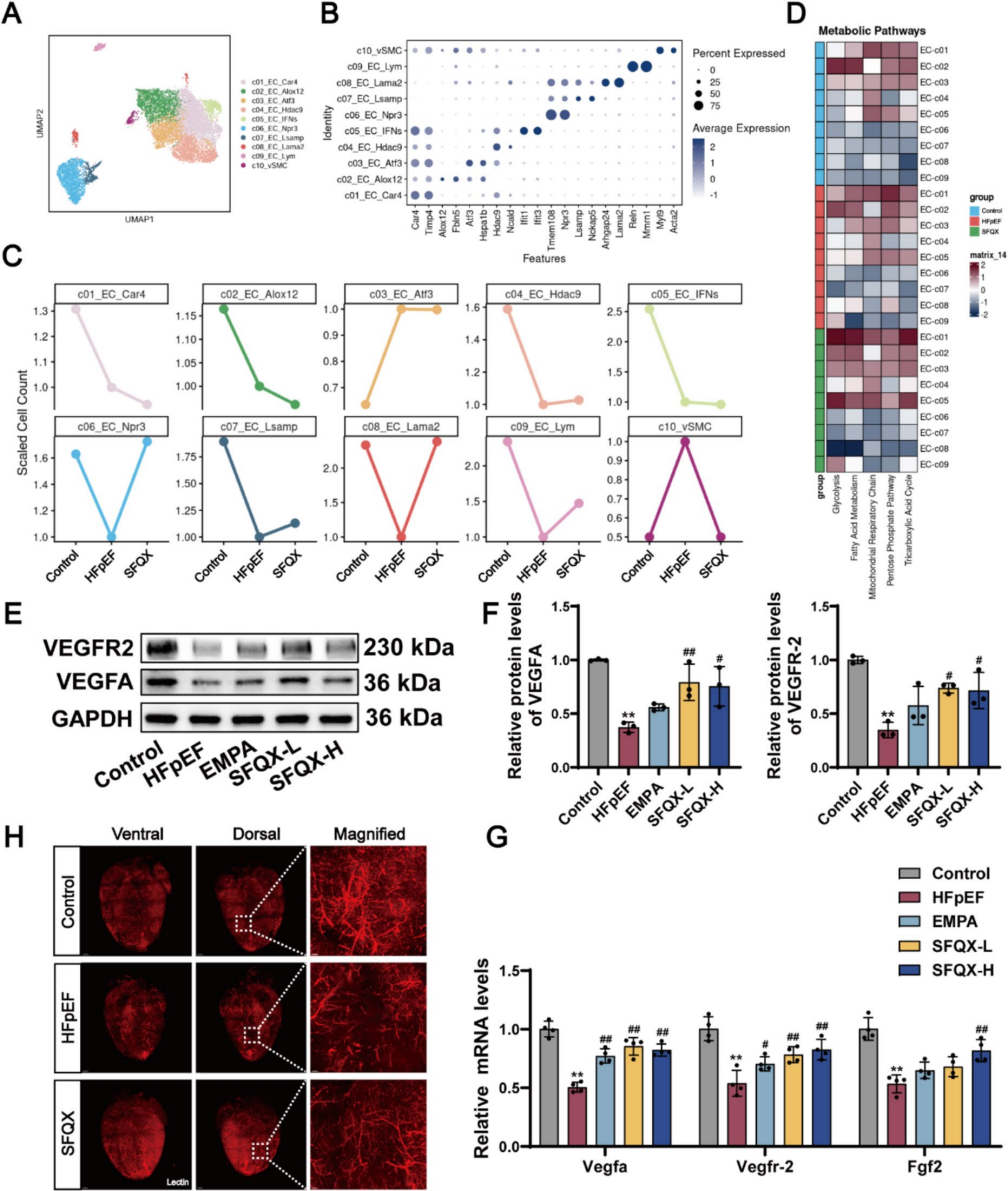
SFQX modulates endothelial cell subsets to improve angiogenic disorders. **A** UMAP embedding of endothelial cells (ECs), colored by manually annotated subsets. **B** Marker genes for each EC cluster. **C** Abundance of EC subsets in Control, HFpEF, and SFQX groups. **D** Heatmap of average expression levels for key genes in multiple energy metabolism pathways. **E**, **F** Western blot analysis of VEGFA and VEGFR2 protein expressions (n = 3). **G** qPCR analysis of *Vegfa*, *Vegfr-2*, and *Fgf2* mRNA expressions (n = 4). **H** Cardiac microvascular network visualized by lectin staining. Data are presented as mean ± SD. *p < 0.05, **p < 0.01 vs. Control group. ^*#*^*p* < 0.05, ^*##*^*p* < 0.01 vs. HFpEF group

Endothelial dysfunction plays a pivotal role in the pathogenesis and progression of HFPEF [30]. The 10,808 ECs derived from all samples clustered into ten distinct EC subsets and displayed individual gene expression profiles (Fig. 7A, B). Following differential analysis of each subset, we observed multiple metabolism-related DEGs across several subsets. Therefore, we performed metabolic pathway scoring, which revealed that SFQX enhances EC metabolic activity by promoting multi-substrate utilization, particularly within the c01-c05 ECs clusters (Fig. 7D). In addition, SFQX exhibited heterogeneous regulatory effects on various subpopulations, and an overall increase in EC numbers was observed after SFQX treatment compared with the model group (Fig. 7C). These results suggest that SFQX can reverse the reduction in ECs observed in HFpEF. Next, western blot analysis confirmed that SFQX treatment promoted the expression of angiogenesis markers, including VEGFA and VEGFR2 (Fig. 7E, F). qPCR analysis further revealed a general upregulation in the mRNA expression of key markers, including *Vegfa, Vegfr-2, and Fgf2*, following SFQX treatment (Fig. 7G). The cardiac microvascular network was visualized across the entire heart using lectin perfusion. Notably, SFQX treatment enhanced microvascular formation in HFpEF hearts, consistent with the above findings (Fig. 7H).

### SFQX restores lymphatic endothelial homeostasis to promote cardiac lymphatic drainage

**Fig. 8:**
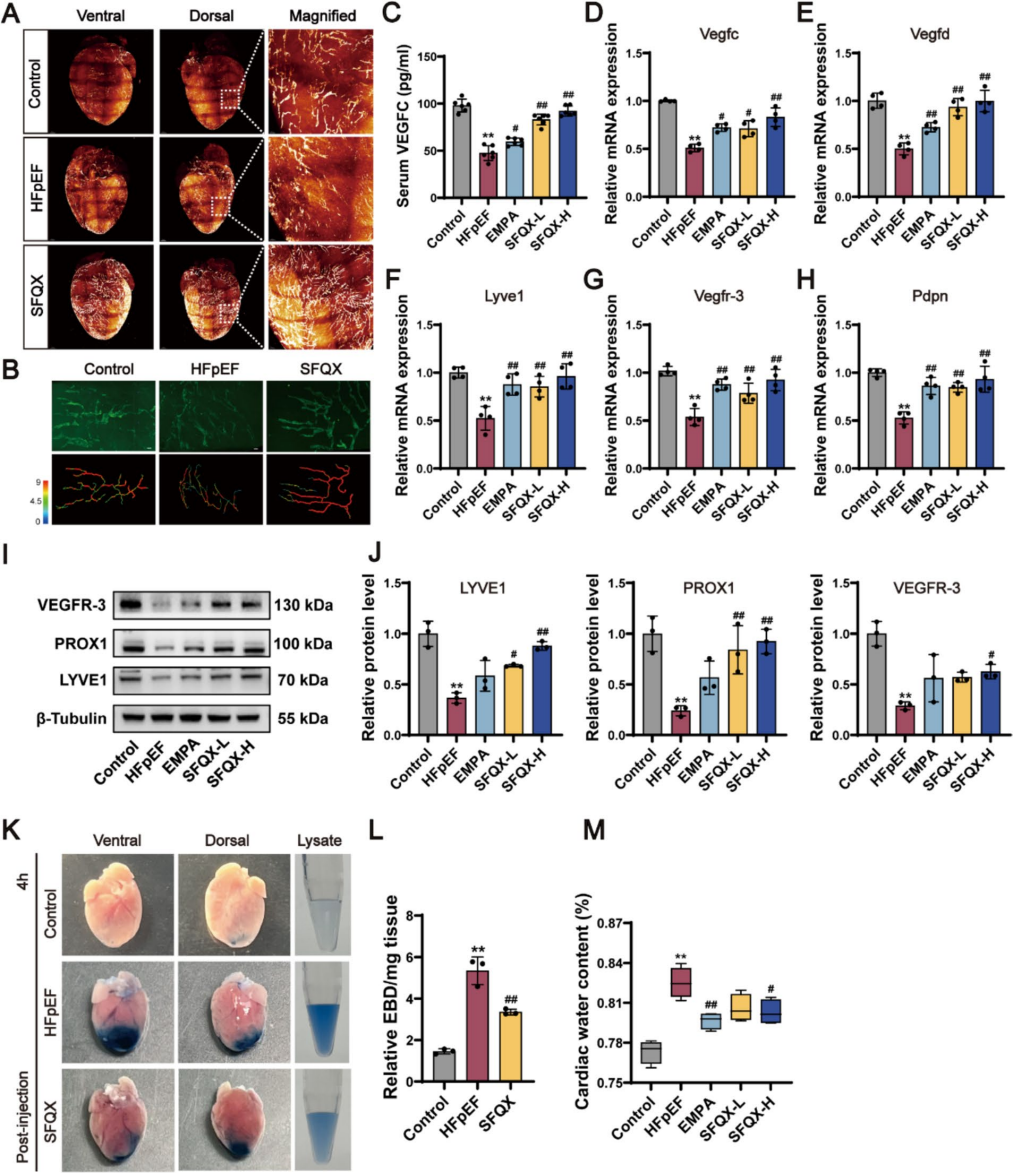
SFQX restores lymphatic endothelial homeostasis to promote cardiac lymphatic drainage. **A** Whole heart clearing and whole-mount immunofluorescence staining for LYVE1 (lymphatic endothelial hyaluronic acid receptor 1). **B** Reconstruction and quantification of the 3D cardiac lymphatic system. **C** The concentration of VEGFC in serum (n = 6). qPCR analysis of **D** *Vegfc*, **E** *Vegfd*, **F** *Lyve1*, **G** *Vegfr-3*, and **H** *Pdpn* mRNA expressions (n = 4). **I–J** Western blot analysis of the protein level of LYVE1, PROX1 and VEGFR-3 in the heart (n = 3). **K**, **L** Assessment of cardiac lymphatic drainage function using Evans blue dye (n = 4). **M** Quantification of cardiac edema by the wet-to-dry heart weight ratio (n = 4). Data are presented as mean ± SD. *p < 0.05, **p < 0.01 vs. Control group. ^*#*^*p* < 0.05, ^*##*^*p* < 0.01 vs. HFpEF group

Insufficient lymphatic function and/or impaired lymphangiogenesis lead to fluid accumulation and the development of edema. Our scRNA-seq analysis showed that SFQX treatment increased the proportion of lymphatic endothelial cells (LECs) in the hearts of HFpEF mice (Fig. 7C), suggesting that lymphatic endothelial remodeling may represent an important component of its endothelial regulatory effects. Given this observation, we next examined whether SFQX was associated with restoration of lymphatic endothelial structure and function in the HFpEF heart. We performed whole-heart immunofluorescence staining against LYVE1 (a specific lymphatic endothelial cell marker) and conducted imaging on a light-sheet fluorescence microscopy platform to enable 3D visualization of the entire lymphatic network (Fig. 8A). Compared to the control group, HFpEF mice exhibited significant structural abnormalities in cardiac lymphatics, characterized by sparsity, reduced vessel volume, discontinuity, and fragmentation. Notably, SFQX treatment markedly ameliorated these pathological structural alterations (Fig. 8A, B). We also found that SFQX treatment increased the serum level of VEGFC (Fig. 8C). Besides, SFQX significantly upregulated the mRNA levels of key LEC markers and regulatory genes, such as *Vegfc, Vegfd, Lyve1, Vegfr3* and *Pdpn* (Fig. 8D–H). Correspondingly, SFQX treatment also led to a significant increase in the protein levels of key LEC markers, including LYVE1, VEGFR3, and PROX1, in HFpEF mice (Fig. 8I, J). Together, these findings suggest that SFQX treatment is associated with molecular changes consistent with lymphatic endothelial remodeling. Next, we evaluated the capacity of cardiac lymphatic drainage by direct injection of Evans blue dye into the cardiac apex. SFQX treatment significantly enhanced dye clearance, indicating improved lymphatic transport function (Fig. 8K, L). Consistent with improved drainage, SFQX administration also ameliorated cardiac edema, as evidenced by a reduced heart wet-to-dry weight ratio (Fig. 8M). Collectively, these results indicate that SFQX treatment is associated with restoration of lymphatic endothelial homeostasis, improved cardiac lymphatic drainage, and reduced fluid retention in HFpEF. However, the associations described above do not establish a causal role for lymphatic endothelial remodeling in mediating the therapeutic effects of SFQX and require further functional validation.

In addition, we used SwissTargetPrediction to predict the potential targets of 110 active compounds in SFQX, and retained predicted targets with Probability > 0 as the candidate target set. We then screened DEGs reversed after SFQX treatment based on scRNA-seq, namely SFQX rescued genes, using the criteria of adjusted P value < 0.05 and |log2FC|> 0.585. Finally, Fisher’s exact test was used to evaluate the significant overlap between candidate compound targets and cell-type-specific SFQX rescued genes. Based on the overlap enrichment results, we inferred the potential associations between candidate compounds and specific cell types. These associations were not functionally validated in the present study. For example, aloe-emodin, adenosine, and polyporusterone B were linked to endothelial cells (ECs), whereas quercetin, umbelliferone, and scopoletin were associated with fibroblasts (FBs) (Fig. S2, Table S4). These results suggest that several bioactive components may preferentially regulate distinct cardiac cell types in HFpEF.

## Discussion

HFpEF is increasingly recognized as a highly heterogeneous clinical syndrome rather than a uniform disease entity [7]. This heterogeneity is reflected not only in patient phenotypes and comorbidities but also at the cellular and molecular levels within the heart. Accumulating evidence indicates that HFpEF progression is driven by maladaptive changes in the composition, functional states, and interactions of multiple cardiac cell populations, rather than by dysfunction of a single dominant cell type or pathway [8, 10, 11]. In this context, therapeutic strategies capable of coordinately modulating pathological cellular heterogeneity may be particularly advantageous for HFpEF.

In the present study, we demonstrate that SFQX exerts robust therapeutic effects in a murine HFpEF model, which is achieved by remodeling disease-associated cellular heterogeneity across multiple cardiac lineages and restoring suppressed cell–cell communication under HFpEF pathological conditions. By integrating scRNA-seq with functional and molecular validation, our data reveal that SFQX does not simply suppress individual pathological processes but instead reprograms aberrant cell states within immune, stromal, and endothelial compartments. This systems-level modulation of cellular heterogeneity provides a mechanistic explanation for the broad efficacy of SFQX in a disease characterized by multifactorial pathophysiology.

A central finding of this study is that HFpEF is associated with a pronounced shift toward maladaptive immune cell states, particularly within the macrophage subpopulation. Classical monocytes in the circulation are recruited to sites of injury, infiltrate the heart, and differentiate into macrophages, thereby exacerbating inflammation and fibrosis [31]. Single-cell analysis revealed an expansion of monocyte-derived pro-inflammatory macrophage subsets, accompanied by a relative loss of tissue-resident homeostatic macrophages. Such alterations are consistent with the concept that chronic metabolic stress and low-grade inflammation drive persistent immune activation in HFpEF [10]. SFQX treatment markedly attenuated this imbalance by reducing the abundance of inflammatory macrophage subsets, suppressing pro-inflammatory transcriptional programs, and redirecting macrophage differentiation trajectories toward resident-like states. The shift toward a homeostatic phenotype helps restrain excessive inflammation, promote its resolution, and ultimately attenuate myocardial injury [26]. Importantly, these changes were accompanied by systemic and cardiac reductions in inflammatory mediators, supporting the notion that SFQX restores immune homeostasis by correcting pathological immune cell-state polarization rather than broadly inhibiting immune function.

Beyond immune dysregulation, aberrant fibroblast activation represents a key cellular hallmark of HFpEF [32, 33]. Fibroblasts in HFpEF hearts exhibit marked heterogeneity. Among them, the myofibroblast‑like subset shows an activated state accompanied by increased ECM production [34]. Meanwhile, the pro‑inflammatory/chemokine‑secreting subset recruits immune cells and amplifies local pro‑fibrotic signaling through the release of CCL‑family chemokines [8, 11]. These fibroblast states are likely induced by sustained inflammatory and metabolic cues, thereby directly contributing to myocardial stiffening and diastolic dysfunction [11]. Our single-cell data demonstrate that SFQX selectively suppresses profibrotic FB subsets and downregulates fibrosis-associated gene programs, including those involved in ECM deposition, cytoskeletal remodeling, and cell–matrix adhesion. These transcriptional changes were corroborated by histological and biochemical evidence of reduced interstitial and perivascular fibrosis. Collectively, these findings indicate that SFQX mitigates cardiac fibrosis by normalizing fibroblast state heterogeneity rather than indiscriminately depleting fibroblasts.

Endothelial cells also displayed marked heterogeneity in HFpEF, encompassing diverse metabolic and functional states associated with microvascular dysfunction [35, 36]. HFpEF hearts showed a reduction in endothelial abundance and impaired metabolic flexibility across EC subsets, consistent with previous reports linking endothelial dysfunction to impaired myocardial perfusion and inflammation [37]. SFQX treatment increased endothelial cell representation and enhanced metabolic pathway activity across multiple EC subsets, suggesting improved endothelial adaptability to metabolic stress. Concomitantly, SFQX promoted angiogenic signaling and restored microvascular density, indicating functional recovery of the endothelial compartment.

Within the heterogeneous endothelial compartment, lymphatic endothelial cells emerged as a particularly functionally relevant subset in the context of HFpEF. Although less abundant than blood vascular endothelial cells, LECs play a critical role in maintaining cardiac homeostasis by facilitating the clearance of excess fluid, proteins, metabolic waste, and immune cells from the cardiac interstitium [38–40]. This function may be conceptually consistent with the TCM principle of “draining dampness,” which emphasizes the elimination of pathological substances from the body [41]. Cardiac lymphatic vessels form an intricate network closely associated with the coronary vasculature, thereby promoting interstitial fluid drainage and tissue fluid balance [42]. Structural or functional abnormalities in LECs may result in interstitial fluid retention and the accumulation of macromolecules and metabolic waste products [43]. From a TCM perspective, these changes may provide a possible biological correlate for “phlegm-dampness” [41] and may contribute to impaired cardiac diastolic function through persistent inflammation and disturbances in lipid metabolism [38]. Our single-cell analysis identified a marked reduction in LEC representation and lymphangiogenic gene programs in HFpEF hearts, indicating that lymphatic endothelial dysfunction represents an integral component of endothelial heterogeneity remodeling during disease progression.

Importantly, SFQX treatment selectively expanded the LEC fraction and reinstated their transcriptional profile within the endothelium, without disrupting overall endothelial identity. This targeted normalization suggests that SFQX does not indiscriminately stimulate endothelial proliferation but rather rebalances endothelial subpopulations toward a more homeostatic composition. Clinical and experimental studies have demonstrated that HFpEF exhibited structural abnormalities in cardiac lymphatics, characterized by sparsity, reduced lymphatic volume and fragmented vessels [44, 45]. However, the promotion of lymphangiogenesis through VEGFC or other stimulatory approaches can effectively ameliorate cardiac hypertrophy, fibrosis, edema, and inflammatory responses [39, 44]. Functional validation further demonstrated that restoration of LEC states was accompanied by enhanced lymphatic transport capacity and reduced myocardial edema, providing a modern biological explanation for the "promoting fluid excretion" effect of SFQX. These findings support a close functional association between lymphatic endothelial remodeling and tissue-level improvement after SFQX treatment, while the causal role of this axis remains to be determined in future loss-of-function studies.

From a broader perspective, these findings position LECs as a critical interface through which immune, stromal, and vascular remodeling converge in HFpEF [38]. By facilitating the clearance of excess interstitial fluid and inflammatory mediators, LECs may indirectly attenuate fibroblast activation and sustain a less pro-inflammatory myocardial microenvironment [45]. Thus, the recovery of LEC homeostasis represents a functionally important cellular event within the broader process of SFQX-mediated remodeling of cardiac cellular heterogeneity. This observation highlights the need to consider functionally specialized endothelial subpopulations in evaluating therapies aimed at restoring myocardial homeostasis in HFpEF.

Taken together, these cell-type-specific effects reveal a unifying principle: SFQX ameliorates HFpEF by coordinately reprogramming maladaptive cellular states across interconnected cardiac cell populations. Immune, stromal, and endothelial cells do not operate independently in HFpEF. Instead, they form an interdependent cellular network in which inflammatory activation, fibrotic remodeling, and vascular dysfunction reinforce one another [46]. By simultaneously dampening inflammatory macrophage polarization, restraining fibroblast activation, and restoring endothelial functional diversity, SFQX disrupts this pathological feed-forward loop and promotes a more homeostatic myocardial microenvironment.

From a therapeutic perspective, these findings underscore the limitations of single-target interventions in HFpEF and highlight the potential advantages of multi-component therapies capable of network-level regulation. TCM formulations such as SFQX are inherently designed to act on multiple biological processes, which may explain their ability to modulate complex cellular ecosystems [47]. Our study provides single-cell level evidence supporting the concept that such therapies can reshape pathological cellular heterogeneity, offering a mechanistic framework that bridges ene holistic principles with modern systems biology.

Several limitations of this study should be acknowledged. First, although scRNA-seq enables high-resolution characterization of cardiac cellular heterogeneity, it captures cellular states at a single time point and therefore cannot fully resolve the dynamic trajectories through which SFQX remodels cell states and intercellular interactions during HFpEF progression. Second, although our data indicate coordinated remodeling across multiple cardiac cell populations, it remains unclear to what extent SFQX acts directly on specific cell types versus indirectly through microenvironmental changes. Third, rescue or loss-of-function experiments, such as VEGFR3 pathway blockade, were not performed to determine whether the therapeutic effects of SFQX are causally dependent on lymphatic endothelial homeostasis. In addition, the present study does not establish a direct correlation between individual compounds and specific cellular alterations in HFpEF, and the observed effects may reflect the coordinated actions of multiple compounds rather than a single dominant constituent. Further experimental and clinical studies are therefore required to validate the predicted bioactive networks and determine whether comparable remodeling of cardiac cellular heterogeneity occurs in human HFpEF patients treated with SFQX.

**Fig. 9:**
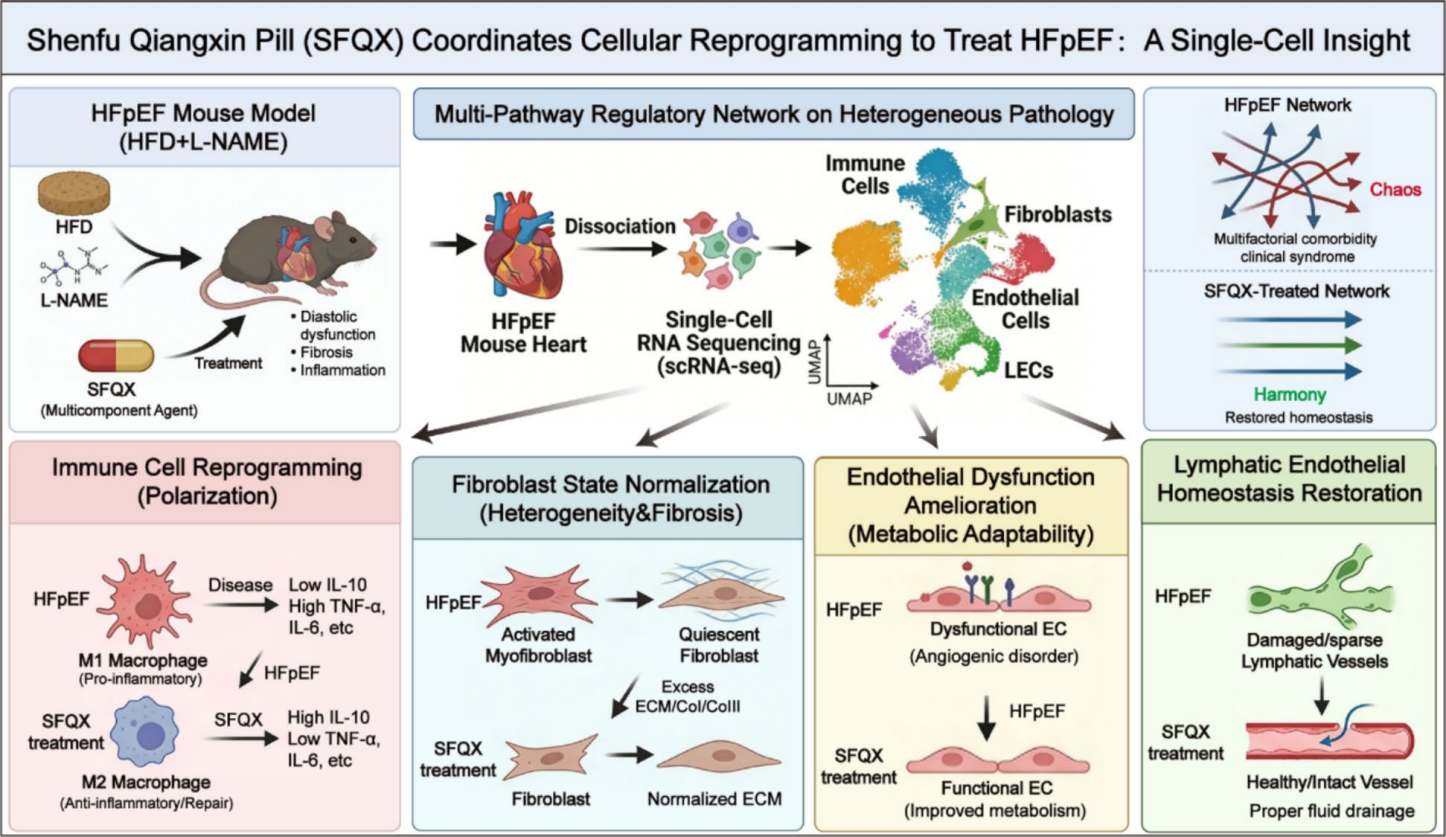
Schematic diagram of SFQX modulating cardiac cell heterogeneity for the treatment of HFpEF

In conclusion, SFQX alleviates HFpEF by reprogramming pathological cellular heterogeneity across immune, stromal, and endothelial compartments of the heart. By restoring balanced cell states and intercellular homeostasis, SFQX appears to modulate pathological multicellular crosstalk in a manner consistent with the holistic therapeutic properties of traditional Chinese medicine (Fig. 9). Rather than defining a single active constituent, our study delineates the potential regulatory networks of bioactive compounds across cardiac cell populations. These findings provide mechanistic insight into the systems-level actions of SFQX and highlight cellular heterogeneity remodeling as a promising treatment strategy for complex heart failure.

## Supplementary Information

Supplementary  Material 1

Supplementary Material 2

Supplementary Material 3

Supplementary  Material 4

Supplementary  Material 5

Supplementary  Material 6

Supplementary Material  7

## Data Availability

The data produced from this study will be made available from the corresponding author on reasonable request.

## Abbreviations

ANP: Atrial natriuretic peptide
CHF: Chronic heart failure
DEGs: Differentially expressed genes
EC: Endothelial cell
ECM: Extracellular matrix
EMPA: Empagliflozin
FB: Fibroblast
HFD: High-fat diet
HFpEF: Heart failure with preserved ejection fraction
LEC: Lymphatic endothelial cell
L-NAME: Nω-Nitro-L-arginine methyl ester hydrochloride
LVEF: Left ventricular ejection fraction
scRNA-seq: Single-cell RNA sequencing
SFQX: Shenfu Qiangxin pills
UMAP: Uniform Manifold Approximation and Projection
