# Supplementary figures and images for: Single-cell transcriptomics reveals how Shenfu Qiangxin pill ameliorates HFpEF by modulating cardiac cellular heterogeneity
Source: Chin Med. 2026 Jul 20;21:199. doi: 10.1186/s13020-026-01456-3 (PMC13383478; doi:10.1186/s13020-026-01456-3)

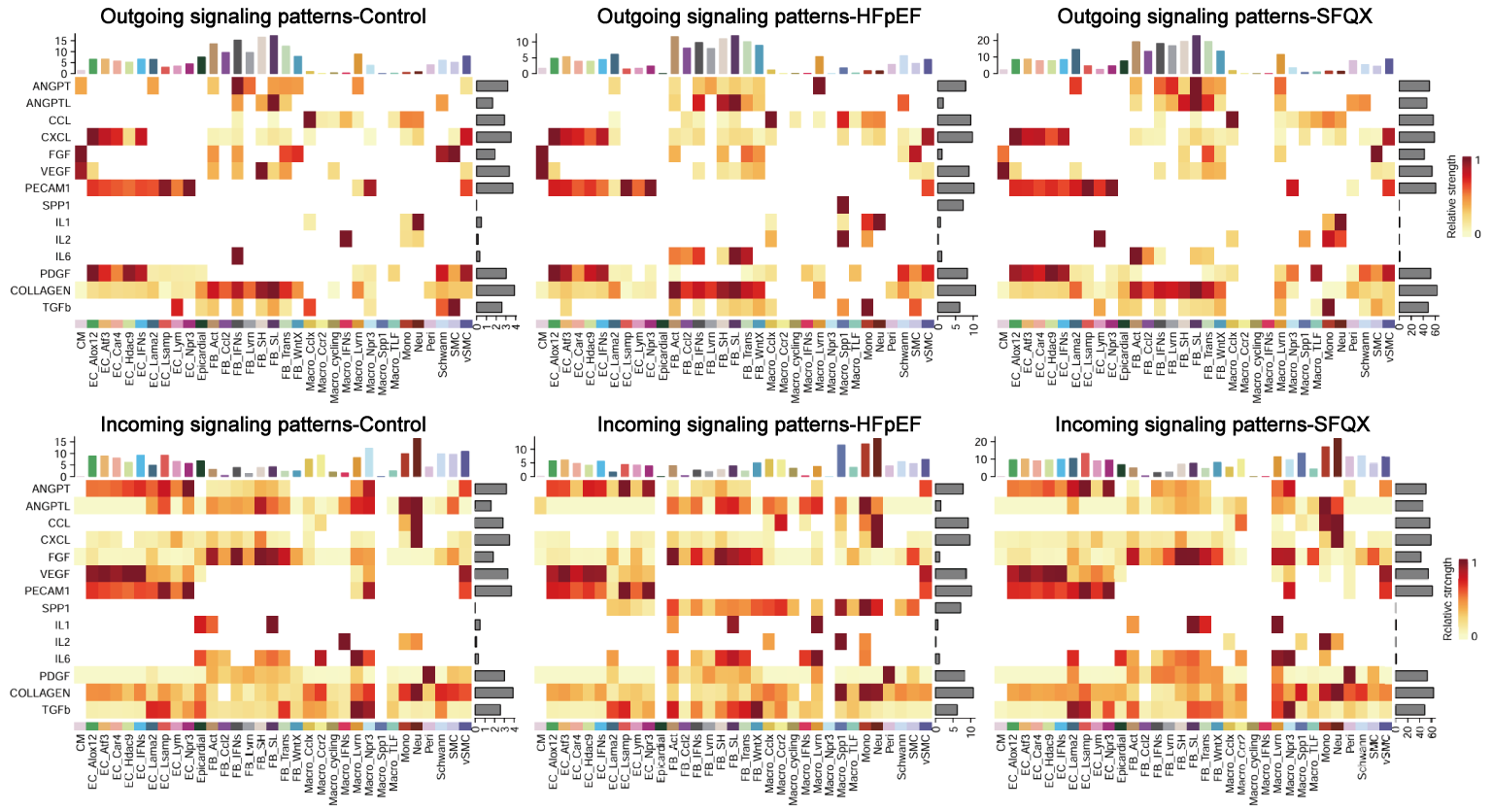

Supplement: Supplementary file 1 — Supplementary Material 1 [file 13020_2026_1456_MOESM1_ESM.tif]

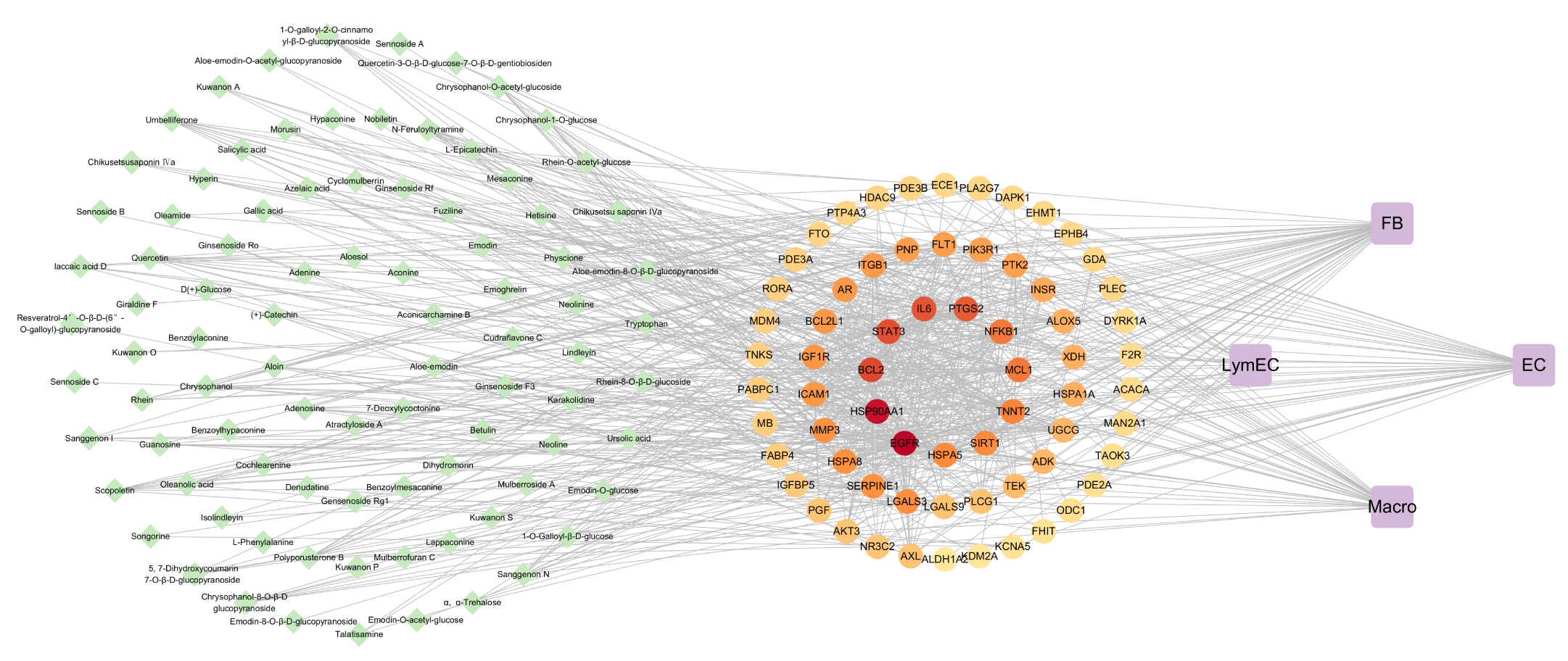

Supplement: Supplementary file 2 — Supplementary Material 2 [file 13020_2026_1456_MOESM2_ESM.tif]

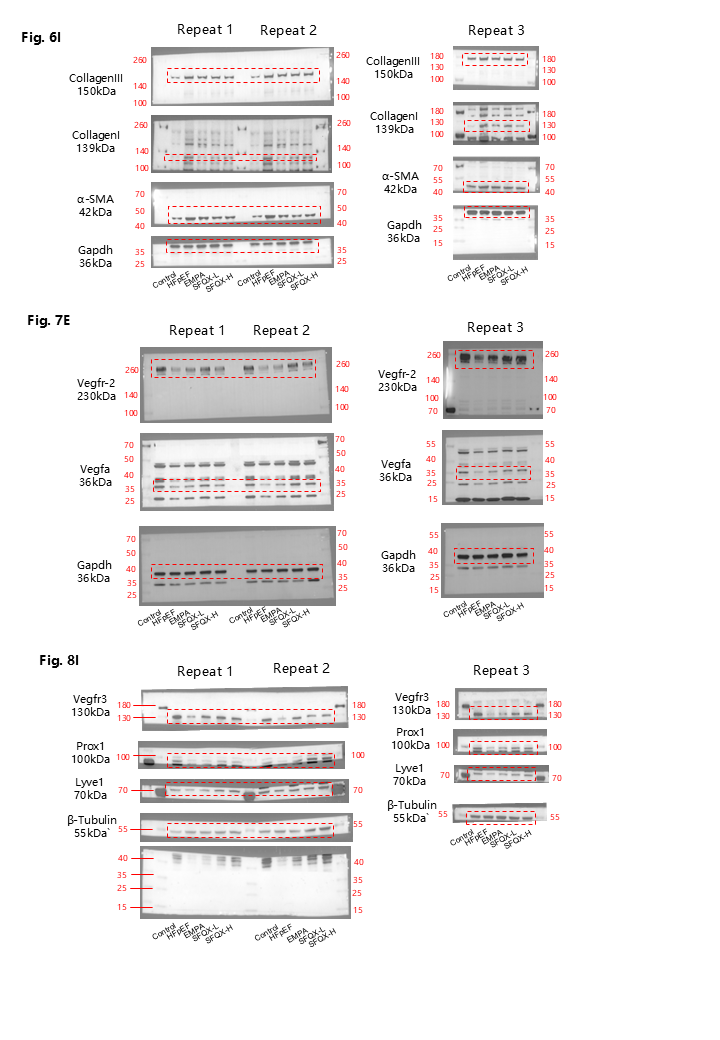

Supplement: Supplementary file 7 — Supplementary Material 7 [file 13020_2026_1456_MOESM7_ESM.tif]
