# Supplementary material for: Single-cell transcriptomics reveals how Shenfu Qiangxin pill ameliorates HFpEF by modulating cardiac cellular heterogeneity
Source: Chin Med. 2026 Jul 20;21:199. doi: 10.1186/s13020-026-01456-3 (PMC13383478; doi:10.1186/s13020-026-01456-3)
Supplement: Supplementary file 3 — Supplementary Material 3 [file 13020_2026_1456_MOESM3_ESM.docx]

| Gene name | Forward Primer | Reverse Primer |
| --- | --- | --- |
| Glut1 | GTGCTCCTCGTGCTCTTCTTCATC | CTCCTCGGGTGTCTTGTCACTTTG |
| Hk-2 | ATGATCGCCTGCTTATTCACG | CGCCTAGAAATCTCCAGAAGGG |
| Pfkm | ATGAATGCCGCTGTTCGCTCTAC | CCAGACCCTCAAAGCCATCATGTAC |
| Pfkfb3 | TCTGTCACCAGGCTGTTCTACG | TGTGCTCACCGATTCTACATTCAAG |
| Pkm | TGCCGCCTGGACATTGACTC | ATTCAGCCGAGCCACATTCATTC |
| Pdk1 | ACTCAACCAGCACTCCTTATTGTTC | GCCTAGCGTTCTCATAGCCATC |
| Acsl1 | TCCTGGACGACTTGTTGAAACTTG | GCCTCTTCGCCTTCAGTGTTG |
| Cpt-1a | CACAACAACGGCAGAGCAGAG | ACACCACATAGAGGCAGAAGAGG |
| Cpt-2 | CCTGCTCGCTCAGGATAAACA | GTGTCTTCAGAAACCGCACTG |
| Acadvl | ACTCACTGGGCTGGGCAATG | CCGATTCCTGTCCTCCGTCTC |
| Hadhb | TCGGGTTTGTTGCATCGGA | GGCCAGAAGCTATCAGACCAA |
| Acaa2 | ATAAGCAGAGAAGACTGTGACAGATAC | CATTGAAGTAGCCAGCCTCGTTAG |
| IL-6 | CTTCTTGGGACTGATGCTGGTGAC | AGGTCTGTTGGGAGTGGTATCCTC |
| IL-1β | TCGCAGCAGCACATCAACAAGAG | AGGTCCACGGGAAAGACACAGG |
| TNF-α | CACGCTCTTCTGTCTACTGAACTTC | CTTGGTGGTTTGTGAGTGTGAGG |
| IFN-γ | CTGGAGGAACTGGCAAAAGGATGG | GACGCTTATGTTGTTGCTGATGGC |
| MCP-1 | CACTCACCTGCTGCTACTCATTCAC | CTTCTTTGGGACACCTGCTGCTG |
| Vegfa | CCACGACAGAAGGAGAGCAGAAG | GGTCTCAATCGGACGGCAGTAG |
| Vegfr-2 | GACTGTGGCGAAGTGTTTTTGA | GTGCAGGGGAGGGTTGGCGTAG |
| Fgf2 | GCGACCCACACGTCAAACTA | CCGTCCATCTTCCTTCATAGC |
| Vegfc | CACTTGCTGTGCTTCTTGTCTCTG | AACTGCTCCTCCAGGTCTTTGC |
| Vegfd | AACACAAGCACCTCCTACATCTCC | GCAAGCACTTACAACCCGTATGG |
| Lyve1 | CTCGTGCAAGACCTTTCCATT | GCCTCGTTGGCTTCTGTGAA |
| Vegfr-3 | CGCAGAGTGATGTGTGGTCCTTC | CCGCTGGCAGAACTCCTCATTG |
| Pdpn | TGTGGACCGTGCCAGTGTTG | CCAGAGGTGCCTTGCCAGTAG |
